# Baicalein Suppresses Stem Cell-Like Characteristics in Radio- and Chemoresistant MDA-MB-231 Human Breast Cancer Cells through Up-Regulation of IFIT2

**DOI:** 10.3390/nu11030624

**Published:** 2019-03-14

**Authors:** So Yae Koh, Jeong Yong Moon, Tatsuya Unno, Somi Kim Cho

**Affiliations:** 1 Interdisciplinary Graduate Program in Advanced Convergence Technology and Science, Jeju National University, Jeju 63243, Korea; soyee.go@jejunu.ac.kr; 2 Subtropical/Tropical Organism Gene Bank, Jeju National University, Jeju 63243, Korea; owenmjy@jejunu.ac.kr (J.Y.M.); tatsu@jejunu.ac.kr (T.U.); 3 Faculty of Biotechnology, College of Applied Life Sciences, SARI, Jeju National University, Jeju 63243, Korea

**Keywords:** baicalein, cancer stem cell, chemoresistance, IFIT2, radioresistance, triple-negative breast cancer

## Abstract

Resistance to both chemotherapy and radiation therapy is frequent in triple-negative breast cancer (TNBC) patients. We established treatment-resistant TNBC MDA-MB-231/IR cells by irradiating the parental MDA-MB-231 cells 25 times with 2 Gy irradiation and investigated the molecular mechanisms of acquired resistance. The resistant MDA-MB-231/IR cells were enhanced in migration, invasion, and stem cell-like characteristics. Pathway analysis by the Database for Annotation, Visualization and Integrated Discovery revealed that the NF-κB pathway, TNF signaling pathway, and Toll-like receptor pathway were enriched in MDA-MB-231/IR cells. Among 77 differentially expressed genes revealed by transcriptome analysis, 12 genes involved in drug and radiation resistance, including interferon-induced protein with tetratricopeptide repeats 2 (IFIT2), were identified. We found that baicalein effectively reversed the expression of IFIT2, which is reported to be associated with metastasis, recurrence, and poor prognosis in TNBC patients. Baicalein sensitized radio- and chemoresistant cells and induced apoptosis, while suppressing stem cell-like characteristics, such as mammosphere formation, side population, expression of Oct3/4 and ABCG2, and CD44^high^CD24^low^ population in MDA-MB-231/IR cells. These findings improve our understanding of the genes implicated in radio- and chemoresistance in breast cancer, and indicate that baicalein can serve as a sensitizer that overcomes treatment resistance.

## 1. Introduction

Breast cancer is the most prevalent cancer type worldwide and the second leading cause of cancer death for women, and over 60% of breast cancer patients receive radiation therapy [1]. Triple-negative breast cancers (TNBCs) are breast cancers that are negative for estrogen receptor (ER), progesterone receptor (PR), and human epidermal growth factor receptor 2 (HER2). While only 15–20% of breast cancer patients are classified as TNBCs, TNBC patients present with factors related to poor prognosis and unfavorable features in histologic grade, tumor size, and metastasis [2,3]. Despite advances in breast cancer treatment, TNBC patients still rely on chemotherapy and radiotherapy for disease management. In particular, radiation therapy plays an important role in the management of invasive breast cancer and can improve the survival rate by preventing the spread of metastases [4]. Previously, signaling pathways, including Wnt/β-catenin signaling and androgen receptor signaling, were investigated as mechanisms of radioresistance in TNBC cells [5,6,7]. Adenosine triphosphate-binding cassette (ABC) transporters, phosphatidylinositol 3-kinase/protein kinase B (PI3K/Akt) signaling, and the epithelial–mesenchymal transition (EMT)-related pathway were reported as primary mechanisms for chemoresistance in TNBC [8,9]. Chemoresistance may occur simultaneously with radioresistance in cancer patients [10], and resistance to both chemotherapy and radiotherapy remains a major obstacle in achieving a durable response in TNBC patients. However, the underlying mechanisms for both radio- and chemoresistance have not been clarified. Therefore, it is necessary to improve our knowledge and the efficacy of chemotherapy and radiotherapy to prevent metastasis and to produce a continuous therapeutic effect.

Cancer stem cells (CSCs), or tumor initiating cells (TICs), are a particular subpopulation of cells in cancers that are capable of self-renewal and differentiation into the heterogeneous lineages of cancer cells that comprise the tumor [11]. Human TNBC MDA-MB-231 cells are known to have higher CSC populations than other breast cancer cell lines [12,13]. CSCs are depicted at the top of the tumor hierarchy, developing chemo- and radioresistance, recurrence, and metastasis [14,15]. Cell surface markers or specific membrane transporters are used to identify CSCs. Cells expressing CD44^high^CD24^low^ and CD133^high^ on their surfaces have been suggested as breast CSCs [16,17]. Furthermore, ABC transporters such as multidrug-resistance-associated protein 1 (MRP1), multidrug-resistance protein 1 (MDR1), and ATP-binding cassette super-family G member 2 (ABCG2) are overexpressed in the CSCs of breast cancer, pumping out chemicals such as anti-cancer drugs or Hoechst 33342 [18].

Baicalein (5,6,7-trihydroxy-2-phenyl-4H-1-benzopyran-4-one) is an active compound of the roots of *Scutellaria baicalensis* Georgi, a traditional medicinal herb [19]. It is known for its biological benefits in reducing inflammation, tumor progression, and fibrosis, as well as targeting the tumor microenvironment [20,21,22]. Baicalein targets TNBC cells by inducing endoplasmic reticulum stress or changing mitochondrial membrane potentials by inducing intra-cellular reactive oxygen species (ROS) in the caspase-dependent pathway [23] or down-regulating special AT-rich sequence binding protein 1 (SATB1) and the Wnt/β-catenin pathway [24]. In resistant cancer cells, baicalein induced apoptosis by increasing death receptor 5 (DR5) in colon cancer expression [25]. However, the effect of baicalein on treatment-resistant breast cancer cells has not been studied.

In this study, to identify the genes involved in the treatment resistance of TNBC cells and to assess the efficacy of phytochemicals that can overcome treatment resistance, we established and investigated the radio- and chemoresistant TNBC MDA-MB-231/IR cell line. We explored the mechanism underlying baicalein’s inhibition of the viability of treatment-resistant TNBC MDA-MB-231/IR cells and the possibility that baicalein can be a sensitizer to radiation and drugs for TNBC patients with therapy resistance.

## 2. Materials and Methods

### 2.1. Reagents

Dulbecco’s modified Eagle’s medium (DMEM), fetal bovine serum (FBS), insulin, bovine serum albumin (BSA), βFGF, EGF, B-27 supplement, 100 × trypsin/EDTA, 10 × streptomycin/antibiotics, and TRIzol were purchased from Gibco (Gaithersburg, MD, USA), except for TRIzol (Ambion, Austin, TX, USA). Baicalein, luteolin, myricetin, kaempferol, rutin, quercetin, HEPES, Adriamycin (doxorubicin), propidium iodide (PI), Hoechst 33342 dye, 2′,7′-dichlorofluorescin diacetate (H_2_DCF-DA), and RNase A were purchased from Sigma Chemical Co. (St. Louis, MO, USA). The reverse transcription system kit was purchased from Promega (Madison, WI, USA). TOPreal qPCR preMIX was obtained from Enzynomics (Daejeon, Korea). Annexin V-FITC apoptosis detection kit, MitoScreen (JC-1) kit, and Matrigel Matrix were purchased from BD Biosciences (Franklin Lakes, NJ, USA). Cisplatin was obtained from Santa Cruz Biotechnology (Dallas, TX, USA). Dimethyl sulfoxide (DMSO) and 3-(4,5-dimethylthiazol-2-yl)-2,5-diphenyl-tetrazolium bromide (MTT) were obtained from Amresco (Solon, OH, USA). The BCA protein assay kit was purchased from Thermo Fisher Scientific, Pierce Protein Biology (Rockford, IL, USA). Primary antibodies were purchased from Cell Signaling (Danvers, MA, USA), except for IFIT2 (Santa Cruz Biotechnology, Dallas, TX, USA) and actin (Sigma Aldrich, St. Louis, MO, USA). Secondary antibodies were obtained from Vector Laboratories (Burlingame, CA, USA). PVDF membrane was obtained from Millipore (Billerica, MA, USA). The BS ECL Plus kit and 10× phosphate-buffered saline (PBS) were purchased from Biosesang (Seongnam, Gyeonggi, Korea).

### 2.2. Cell Culture and Generation of Resistant Cells 

MDA-MB-231 cells and the derived MDA-MB-231/IR cells were cultured in DMEM supplemented with 10% FBS and 1% streptomycin/antibiotics, and incubated at 37 °C in a humidified incubator (HERAcell 150i, Thermo Fisher, Rockford, IL, USA) with 5% CO_2_. After subculture, when cell confluency reached 70–80%, irradiations were performed. Irradiation was performed at the Applied Radiological Science Institute in Jeju National University using a ^60^CO Theratron-780 teletherapy (MDS Nordion, Ottawa, ON, Canada) unit at a dose rate of 1.52 Gy per minute. Twenty-five cycles of 2 Gy irradiation were performed over five weeks, and the surviving cells were named MDA-MB-231/IR cells.

### 2.3. Cell Viability

The viability of MDA-MB-231 cells and MDA-MB-231/IR cells after sample treatment was determined by MTT assay. Briefly, cells were cultured in 96-well plates at an initial density of 1 × 10^4^ cells/mL in 200 μL per well. During radiation treatment, cells were directly irradiated in a 15-mL conical tube and seeded for 4 days. After the indicated time, the medium was removed, and 100 μL of MTT solution (1 mg/mL) was added; the formazan converted from MTT was dissolved in 150 μL of DMSO. Absorbance was detected by a microplate reader (Tecan, Männedorf, Zürich, Switzerland) at 570 nm.

### 2.4. Clonogenic Assay

The colony formation assay was performed to calculate the surviving fraction. First, 1 × 10^4^ cells were prepared for irradiation, which was performed at the Applied Radiological Science Institute in Jeju National University under the control of a radiologist. Cells (2 × 10^2^) were irradiated by 0, 2, 4, 6, and 8 Gy of gamma rays and seeded on a 12-well plate. After 5 days of incubation, colonies on the plate were washed twice with PBS, fixed, and dyed. Colonies were counted using the NIST integrated colony enumerator (NICE) software program (ftp://ftp.nist.gov/pub/physics/mlclarke/NICE).

### 2.5. Mammosphere Assay

Mammosphere assay was performed according to a previous study [26]. For the mammosphere formation assay, serum-free DMEM supplemented with 1% BSA, 1 μM insulin, 10 ng/mL bFGF, 20 ng/mL EGF, and B-27 supplement was used. Cells were collected by trypsin and washed twice in PBS. Then, 2 × 10^2^ cells were counted and seeded in a non-coated 24-well plate with the stem cell medium described above. Cells were incubated for 1 week to form mammospheres, and the number of mammospheres comprising ≥ 50 cells was counted.

### 2.6. Wound Healing Assay

Cells were plated at 5 × 10^5^ cells per well and incubated in a 6-well plate for 24 h to form a confluent monolayer. A scratch in the shape of a cross was created using the tip of a sterilized 1000-μL pipette. Cells were washed twice with PBS, and the wells were refilled with new DMEM supplemented with 5% FBS. The width of the gap created by the scratch was measured under a microscope immediately after scratching (0 h) and after 24 h.

### 2.7. Invasion Assay

A transwell system (24-well plate, Corning, Cambridge, MA, USA) was used for the invasion assay. The upper inserts of the transwell were filled with 100 μL of 10% Matrigel solution diluted in cold serum-free DMEM. Cells were prepared at a density of 1 × 10^5^ cells per well, washed twice in PBS, and transferred to the upper well of the transwell system in serum-free DMEM. The bottom well was filled with 600 μL of 2% FBS-DMEM. After 24 h of incubation, cells were removed and washed from the insert, except for the cells bound to the membrane. The membrane-bound cells were fixed with 1:1 acetone:methanol fixative and dyed with crystal violet. The stained invading cells were observed under a microscope and counted using the ImageJ program (National Institutes of Health, Bethesda, MD, USA), and the invasive rate of MDA-MB-231/IR cells was compared to that of MDA-MB-231 cells.

### 2.8. Flow Cytometry

A FACSCalibur flow cytometer (BD Biosciences, Franklin Lakes, NJ, USA) was used for flow cytometry analysis. The same number of cells (1 × 10^4^) were detected by FACS. Cells (2 × 10^5^) were detached by trypsin after incubated for 24 h. For cell cycle analysis, cells were washed twice with PBS and fixed in 70% ethanol. Two hours before analysis, cells were treated with RNase A (25 ng/mL) and PI (final concentration of 40 μg/mL) diluted in 500 μL of 2 mM EDTA-PBS and incubated at 37 °C to stain DNA. To detect apoptosis phenomena, the annexin V-FITC Apoptosis Detection Kit I was used following the manufacturer’s protocol. Briefly, cells were washed twice with PBS and mixed with both annexin V-FITC (1:20 dilution in 1× binding buffer) and propidium iodide (PI) (1:50 dilution in 1× binding buffer) for 15 min at room temperature. The analysis was performed within 30 min after the reaction. To detect cell surface markers, CD44-FITC and CD24-PE conjugated dyes were used for analysis. The conjugated dyes were diluted (1:10 dilution in 1× stain buffer) and incubated with cells at 4 °C for 15 min. The side population (SP) was observed with Hoechst 33342 dye. After being detached from the plate, cells were diluted in DMEM buffered with 1 mM HEPES, 2% FBS, and 5 mM Hoechst 33342 at 37 °C for 2 h. The MitoScreen (JC-1) kit was used following the manufacturer’s protocol to detect mitochondrial membrane potential. Briefly, cells were incubated with JC-1 dye (1:100 dilution in 1× assay buffer) at 37 °C for 15 min. After labeling, cells were washed, centrifuged, and resuspended in 500 μL of PBS.

### 2.9. Western Blot Assay

Cells were prepared at a density of 1 × 10^5^ on a 60-mm plate. After incubation or treatment, cells were lysed with RIPA lysis buffer. Most primary antibodies were used at 1:1000 dilutions, except GAPDH (1:10,000), and secondary antibodies were used at 1:5000 dilutions. Protein bands were detected using the BS ECL Plus kit.

### 2.10. Transcriptomic Analysis

Transcriptomic analysis was performed according to a previous study [27]. Total RNA was extracted from human breast cancer cells using TRIzol. RNA (1 µg) was used to construct a library using the Illumina TruSeq mRNA Sample Prep kit (Illumina, San Diego, CA, USA). Poly-T oligo-attached magnetic beads were used to purify mRNA molecules. RNA sequencing (RNA-seq) was performed by Macrogen (Seoul, Korea) according to the manufacturer’s instructions. Prior to the transcriptome assembly, duplicate sequences were removed from raw reads using FastUniq, and the human genome GRCh38 was indexed using Spliced Transcripts Alignment to a Reference (STAR). Trinity was used to assemble reads into transcriptomes. Abundance was calculated using RNA sequencing by expectation maximization (RSEM) to determine statistically significant DEGs (*p* < 0.001 and ≥ 2-fold change) with EdgeR. These DEGs were annotated using Trinotate [28] and uploaded to the Kyoto Encyclopedia of Genes and Genomes (KEGG) Automatic Annotation Server (KAAS) to investigate the involved metabolic pathways.

### 2.11. Pathway Analysis

We used the DAVID tool (Version 6.8 [29]) to predict pathways and gene ontology (GO). Functional analysis of DEGs revealed their associated GO molecular processes, biological processes, and cellular components.

### 2.12. Gene Expression Analysis

Gene expression analyses and RFS were analyzed using TCGA, UCSC Xena browser [30] and the Kaplan–Meier plotter [31]. Patients negative for expression of ER and PR and amplification of HER2 were considered TNBC patients. Gene expression was calculated using fragments per kilobase of transcript per million mapped reads (FPKM). Correlations and *p*-values were analyzed using the R program.

### 2.13. Statistical Analysis

Statistical analyses were carried out by one-way ANOVA and t-test using SPSS (IBM Corp., NY, USA); *p*-values < 0.05 were considered to indicate statistical significance and are denoted with an asterisk (*).

## 3. Results

### 3.1. MDA-MB-231/IR Cells Exhibited Increased Radio- and Chemoresistance Compared to Parental Cells

**Figure 1:**
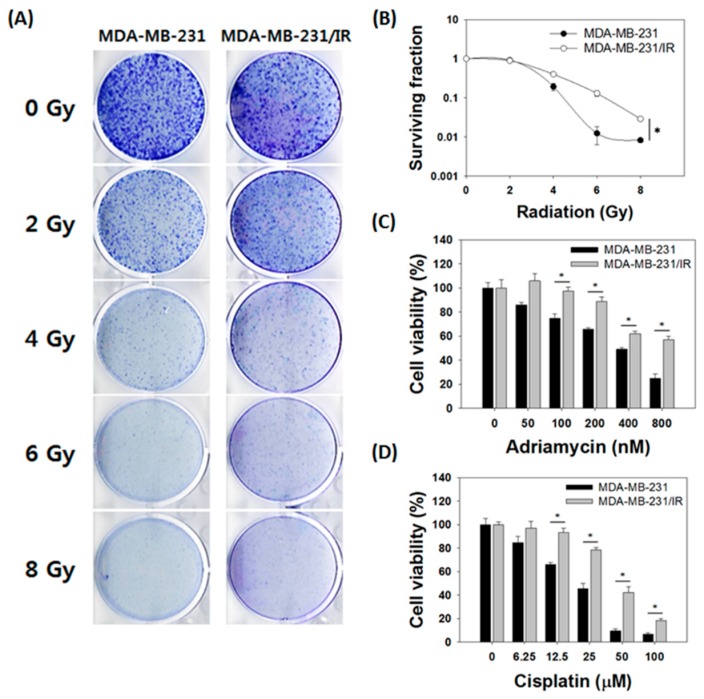
Comparison of parental MDA-MB-231 cells with radio- and chemoresistant MDA-MB-231/IR cells. (**A**) Representative images of the clonogenic assay and (**B**) the surviving fraction of MDA-MB-231 and MDA-MB-231/IR cells after five days of irradiation. MTT assay of MDA-MB-231 and MDA-MB-231/IR cells after (**C**) Adriamycin and (**D**) cisplatin treatment for 24 h. Asterisks (*) indicate significant differences at *p* < 0.05.

To better understand the molecular mechanism of acquired therapy resistance in breast cancer, we generated resistant cancer cells after repetitive 2 Gy irradiations up to a total of 50 Gy. The cells surviving following irradiation were named MDA-MB-231/IR cells. The survival of MDA-MB-231/IR cells after irradiation was significantly higher than that of MDA-MB-231 parental cells, indicating that radiation resistance was obtained in MDA-MB-231/IR cells (Figure 1A). The survival rate of MDA-MB-231/IR cells after irradiation was 1.93, 9.17, and 4.18 times higher than that of MDA-MB-231 parental cells, at 4, 6, and 8 Gy, respectively (Figure 1B). The higher survival of MDA-MB-231/IR cells compared to MDA-MB-231 cells against irradiation was also observed by MTT assay (Supplementary Figure S1A). Taking into account a previous report that chemoresistance may occur simultaneously with radioresistance in cancer patients [10], we also measured the chemoresistance of MDA-MB-231/IR against both Adriamycin (doxorubicin) and cisplatin, which were chosen for their common use in breast cancer therapy. Adriamycin significantly reduced the viability of MDA-MB-231 cells, with an IC_50_ of 393.33 ± 9.43 nM, whereas the IC_50_ in MDA-MB-231/IR cells was > 800 nM (Figure 1C). Cisplatin also reduced viability of MDA-MB-231 cells with an IC_50_ of 22.39 ± 1.61 μM, twofold lower than its IC_50_ in MDA-MB-231/IR cells (46.44 ± 2.82 μM) (Figure 1D). Moreover, both Adriamycin and cisplatin treatment increased the population of sub-G1 cells (Supplementary Figure S1B,C). The population of MDA-MB-231 cells in sub-G1 increased from 2.43 ± 0.52% to 11.87 ± 1.23% after treatment with 100 nM of Adriamycin, whereas in MDA-MB-231/IR cells, the population in sub-G1 increased only from 2.16 ± 0.11% to 3.81 ± 1.39% (Supplementary Figure S1B). Cisplatin treatment increased the sub-G1 population from 4.01 ± 0.28% to 10.91 ± 0.56% in MDA-MB-231 cells, whereas the sub-G1 population did not change in MDA-MB-231/IR cells (Supplementary Figure S1C). This indicates that Adriamycin and cisplatin treatment induced cell death in MDA-MB-231 cells by increasing sub-G1 and apoptosis. These phenomena were also confirmed by annexin V/PI staining (Supplementary Figure S1D,E). In conclusion, these results demonstrate that MDA-MB-231/IR cells were more resistant to Adriamycin and cisplatin treatment than were MDA-MB-231 cells.

### 3.2. Stem Cell Characteristics Were More Prominent in MDA-MB-231/IR Cells than Parent Cells

**Figure 2:**
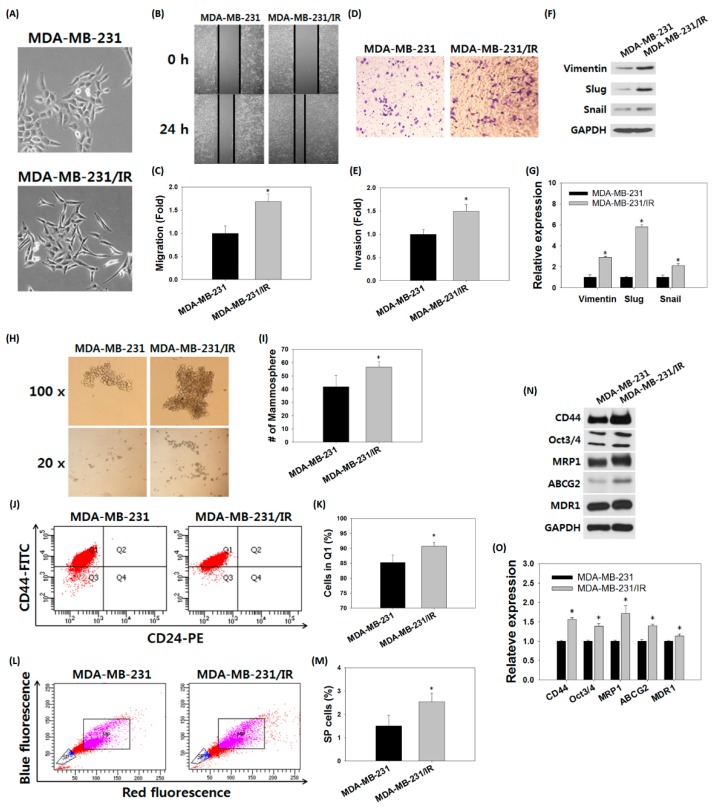
Stem cell-like characteristics of MDA-MB-231/IR cells. (**A**) Morphologies of MDA-MB-231 cells and MDA-MB-231/IR cells. (**B**,**C**) Migration and (**D**,**E**) invasion were analyzed on same number of MDA-MB-231 cells and MDA-MB-231/IR cells for 24 h. (**F**,**G**) Western blot assay for detection of EMT markers expressed in MDA-MB-231 cells and MDA-MB-231/IR cells. GAPDH was used as a control; band intensities were quantified using ImageJ. (**H**,**I**) Mammosphere formation over seven days, (**J**,**K**) expression of cell surface markers, and (**L**,**M**) side population (SP) were detected by FACS analyses. (**N**,**O**) Western blot assay for stem cell markers on MDA-MB-231 cells and MDA-MB-231/IR cells. Asterisks (*) indicate significant differences at *p* < 0.05.

Microscopic observation revealed that the parental MDA-MB-231 cells and the MDA-MB-231/IR cells were clearly differentiated in terms of morphology. MDA-MB-231/IR cells had an especially elongated, spindle-like cell morphology with increased intercellular distance. The morphology of MDA-MB-231/IR cells was altered compared to the parental cells, exhibiting mesenchymal spindle-shaped cells (Figure 2A). We next examined whether the change in morphology in MDA-MB-231/IR cells was related to the EMT. The wound healing assay revealed that 39.55 ± 2.81% of the wound was filled in MDA-MB-231 cells, while 65.85 ± 3.11% of the gap was filled in MDA-MB-231/IR cells (Figure 2B,C). In addition, an invasion assay using a transwell system showed that the number of MDA-MB-231/IR cells that went through the membrane was 1.48 ± 0.13 times greater than that of MDA-MB-231 cells (Figure 2D,E). Moreover, in Western blot analysis, MDA-MB-231/IR cells exhibited higher expression levels of EMT markers such as vimentin, slug, and snail compared to MDA-MB-231 cells (Figure 2F,G). Numerous studies have reported that activation of the EMT program confers CSC properties [32,33,34], therefore, we examined the CSC characteristics of MDA-MB-231/IR cells. The number of mammosphere formations in MDA-MB-231/IR cells was 1.44-fold greater than that of MDA-MB-231 cells when the same number of individual cells was seeded (Figure 2H,I). To scrutinize one aspect of stem cell properties, fluorescence-activated cell sorting (FACS) was utilized to detect the expression of cell surface markers. The FACS analysis demonstrated that the CD44^high^CD24^low^ cell population was higher in MDA-MB-231/IR cells (90.05 ± 1.35%) than in MDA-MB-231 cells (84.98 ± 2.51%) (Figure 2J,K). It is also showed that cells in the side population (SP) could be regarded as stem cells, so we measured the percentage of SP (shown in blue) from the main population (MP; shown in purple) by Hoechst 33342 staining (Figure 2L). The %SP values for MDA-MB-231 and MDA-MB-231/IR cells were 1.52 ± 0.43% and 2.62 ± 0.38%, respectively (Figure 2M), indicating that the %SP in MDA-MB-231/IR cells increased by 1.72 ± 1.13 fold compared to parental MDA-MB-231 cells. In addition, Western blot analysis also demonstrated that the levels of stem cell markers, including CD44, Oct3/4, ABCG2, and MDR1, were increased in MDA-MB-231/IR (Figure 2N,O). In conclusion, in addition to drug resistance and radiation resistance, MDA-MB-231/IR cells had stronger stem cell characteristics than did the parent cells.

### 3.3. Transcriptomic Analysis of MDA-MB-231/IR Cells

**Figure 3:**
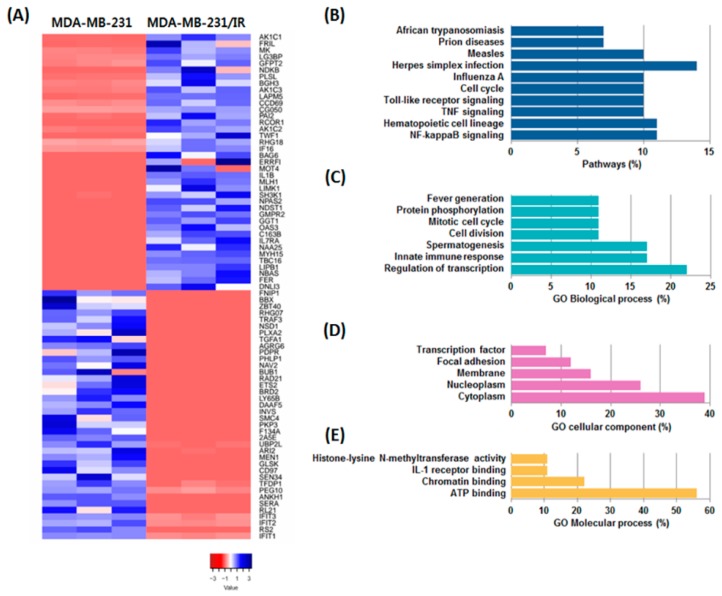
Analyses of differentially expressed genes (DEGs) in MDA-MB-231/IR cells compared to parental MDA-MB-231 cells. (**A**) Heatmap of DEG expression. (**B**) Pathway analysis and (**C**) biological, (**D**) cellular, and (**E**) molecular gene ontology (GO) analyses of DEGs in MDA-MB-231/IR cells.

To better understand chemo- and radioresistance, RNA-seq was performed to identify differentially expressed genes (DEGs). A total of 31,498 transcripts were identified, including 39 up-regulated DEGs and 38 down-regulated genes in MDA-MB-231/IR cells compared to MDA-MB-231 cells (*p* < 0.001) (Figure 3A). After identifying the DEGs, pathways and gene ontology (GO) were analyzed using the Database for Annotation, Visualization and Integrated Discovery (DAVID) software to visualize the gene regulatory network and examine how radiation affected transcription in MDA-MB-231 cell lines. Our analysis revealed that MDA-MB-231/IR cells were enriched in the NF-κB, tumor necrosis factor (TNF), and Toll-like receptor (TLR) signaling pathways compared to MDA-MB-231 cells (Figure 3B). GO biological process analysis revealed the activation of processes including positive regulation of cell division, regulation of transcription from RNA polymerase II promoter, and regulation of protein phosphorylation (Figure 3C). GO cellular component analysis revealed changes in focal adhesion, nucleoplasm, cytoplasm, and membrane activation in experimental groups (Figure 3D). GO molecular process terms indicated that ATP binding, chromatin binding, and interleukin-1 (IL-1) receptor binding were highly regulated in MDA-MB-231/IR cells (Figure 3E). Using the results of the DEG and GO analyses, we identified the enriched pathways to reveal the biological functions and molecular processes involving the up- and down-regulated genes. Literature searches were performed to select genes that play a role in cancer resistance, and 12 genes were identified based on their expression levels and roles in cancer. The expression levels of the 12 selected genes are described by difference and log2 fold change in Table 1. Ten genes, aldo-keto reductase 1C1 (AKR1C1), aldo-keto reductase 1C2 (AKR1C2), aldo-keto reductase 1C3 (AKR1C3), coiled-coil domain-containing 69 (CCDC69), ferritin light chain (FTL), glutamine-fructose-6-phosphate transaminase 2 (GFPT2), galectin-3-binding protein (LG3BP), midkine (MK), transforming growth factor beta induced (TGFBI), and twinfilin actin binding protein 1 (TWF1), were up-regulated in MDA-MB-231/IR cells by up to 6.83 log2 fold, while two genes, IFIT2 and plakophilin 3 (PKP3), were down-regulated. Of the 12 selected genes, six genes, AKR1C1, MK, TGFBI, AKR1C2, TWF1, and PKP3, are reported to function in metastasis, migration, and invasion. Three genes, FTL, AKR1C3, and CCDC69, are reported to be involved in drug resistance, while the remaining selected genes are involved in stem cell-like functions (anti-differentiation), metabolism, and apoptosis. These results imply that the 12 selected genes are likely to be involved in determining the characteristics of MDA-MB-231/IR cells.

### 3.4. Baicalein Treatment Reversed the Level of IFIT2 Expression in MDA-MB-231/IR Cells 

**Figure 4:**
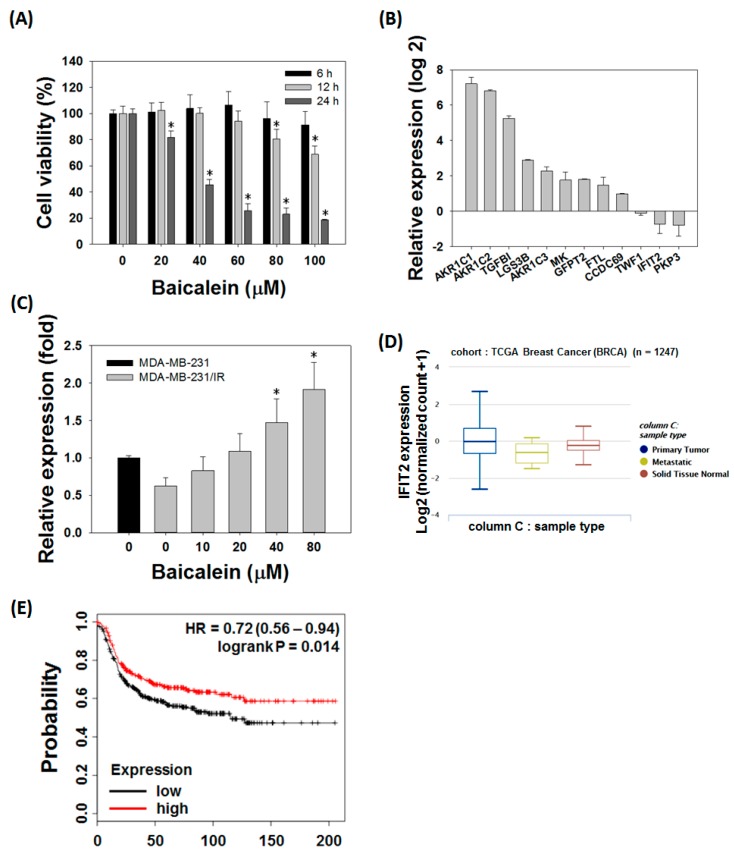
Baicalein treatment reversed levels of IFIT2, which is known to be associated with metastasis and recurrence. (**A**) MTT assay of baicalein on MDA-MB-231/IR cells for 6, 12, and 24 h. (**B**) Expression levels of DEGs involved in resistance in MDA-MB-231/IR cells. (**C**) IFIT2 gene expression after baicalein treatment for 24 h. (**D**) IFIT2 expression in primary tumor, metastatic, and solid normal tissue of breast cancer patients based on Xena browser. (**E**) Relapse-free survival (RFS) plot of TNBC patients analyzed by IFIT2 expression with the Kaplan–Meier plotter. Asterisks (*) indicate significant differences at *p* < 0.05.

There have been compelling reports of polyphenols, flavones, and flavonols that are effective against breast cancer [35,36]. Therefore, we conducted a cell viability test on MDA-MB-231/IR cells against 10 representative anti-cancer phytochemicals: three phenols, three flavones, and four flavonols. Among the compounds tested, the cell-killing effect of baicalein was shown in both MDA-MB-231 cells and the treatment-resistant MDA-MB-231/IR cells, notably showing a lower IC_50_ value in MDA-MB-231/IR cells (Supplementary Table S1). MTT assay revealed that, baicalein effectively induced the death of MDA-MB-231/IR cells in a time- and dose-dependent manner, with an IC_50_ of 38.58 ± 2.86 μM at 24 h (Figure 4A). Baicalein was, therefore, selected for further experiments. We hypothesized that baicalein may target MDA-MB-231/IR cells by regulating the expression of the 12 selected genes identified by transcriptomics. Expression of the 12 genes was analyzed by real-time PCR, and the results showed that their expression levels in MDA-MB-231/IR cells were consistent with the transcriptome analysis, except for TWF1 (Figure 4B). Interestingly, only IFIT2 appeared to be affected by baicalein treatment, and the expression of IFIT2 was reversed in a dose-dependent manner (Figure 4C). To determine the role of IFIT2, we used Xena browser and the Kaplan-Meier plotter to analyze the relationships between IFIT2 expression levels and the progression of The Cancer Genome Atlas (TCGA) breast cancer cohort and the survival rate of TNBC patients. IFIT2 expression was decreased in metastatic breast cancer compared to primary tumor or normal tissue (n = 1247, one-way analysis of variance [ANOVA], *p*-value = 0.0014, f = 6.593) (Figure 4D). The relapse-free survival (RFS) graph analyzed using the Kaplan–Meier plotter revealed that lower IFIT2 expression levels were associated with poor prognosis (lower RFS) compared to TNBC patients expressing higher IFIT2 levels (n = 618, log-rank *p*-value = 0.014, hazard ratio [HR] = 0.72, probe id: 217502-at) (Figure 4E). Taken together, these results indicate that baicalein can target MDA-MB-231/IR cells by reversing IFIT2 expression, which is known to be associated with metastasis and recurrence.

### 3.5. Baicalein Suppressed the Stem Cell-Like Characteristics of MDA-MB-231/IR Cells 

**Figure 5:**
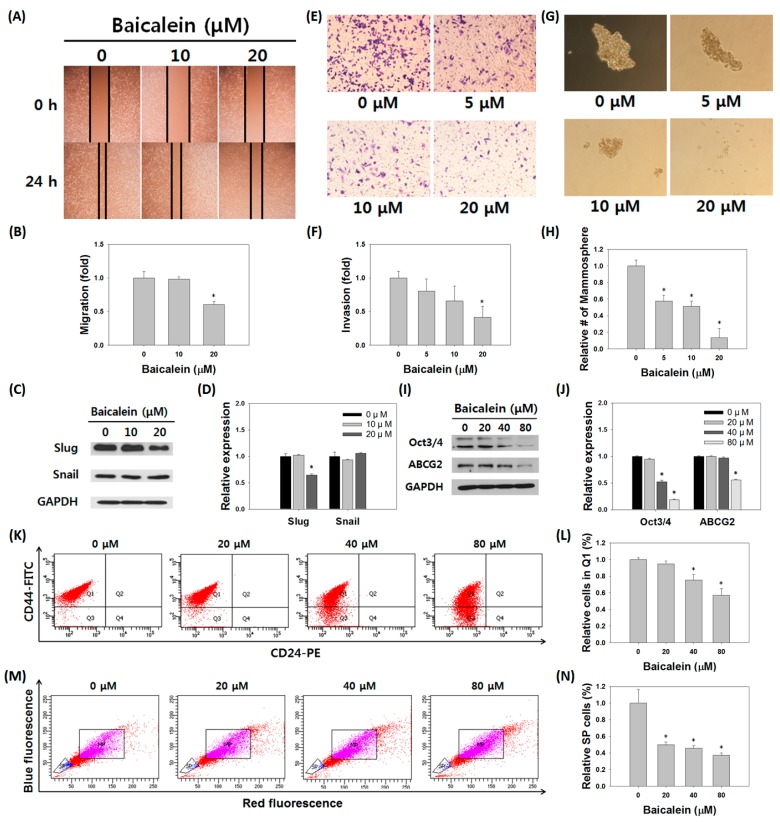
Effects of baicalein on characteristics of MDA-MB-231/IR cells. (**A**,**B**) Migration assay and (**C**,**D**) Western blot analysis of EMT proteins in MDA-MB-231/IR cells were performed after baicalein treatment for 24 h. GAPDH was used as a control; band intensities were quantified using ImageJ. Effects of baicalein on stem cell-like characteristics were determined by (**E**,**F**) invasion assay for 24 h and (**G,H**) mammosphere formation assay for one week. (**I**,**J**) Western blot assay for stem cell markers in MDA-MB-231 cells after baicalein treatment for 24 h. (**K**,**L**) The percentage of expression of cell surface markers and (**M**,**N**) side population (SP) on MDA-MB-231/IR cells were detected by FACS after baicalein treatment for 24 h. Asterisks (*) indicate significant differences at *p* < 0.05.

We tested whether the stem cell-like properties of MDA-MB-231/IR cells could be reversed with increased IFIT2 expression following treatment with baicalein. Western blot, migration, and invasion assays were performed to confirm changes in the EMT phenotype of MDA-MB-231/IR cells. Baicalein treatment decreased migration (0.61 fold) and the expression of slug in MDA-MB-231/IR cells (Figure 5A–D). Invasion ability also decreased (0.43-fold) after baicalein treatment (Figure 5E,F). To further examine the changes in stem cell-like characteristics, mammosphere formation, CD44^high^CD24^low^, and SP population analyses were performed. The size and number of mammospheres and the expression levels of stem cell-like markers were significantly decreased after baicalein treatment (Figure 5G,H). The expression levels of stem cell markers Oct3/4 and ABCG2 decreased following baicalein treatment (Figure 5I,J). In addition, compared to control (baicalein 0 μM), the percentage of CD44^high^CD24^low^ cell population and SP were decreased following baicalein treatment (Figure 5K–N). In summary, baicalein treatment decreased stem cell-like properties in MDA-MB-231/IR cells.

### 3.6. Baicalein Induced Apoptosis and Reversed Radio- and Chemoresistance in MDA-MB-231/IR Cells

**Figure 6:**
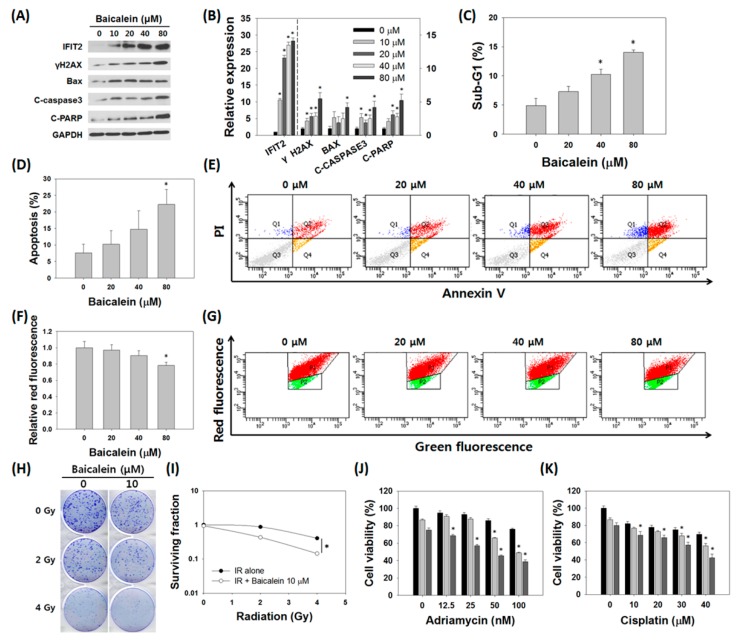
Baicalein induced apoptosis and sensitized resistant MDA-MB-231/IR cells. (**A**,**B**) Expression levels of IFIT2 (left-hand *y*-axis) and apoptosis marker proteins (right-hand *y*-axis) after baicalein treatment for 24 h. (**C**) Cell cycle analysis, (**D**,**E**) annexin V/PI staining, and (**F**,**G**) JC-1 staining were performed to observe induction of apoptosis by baicalein. (**H**,**I**) Baicalein sensitized MDA-MB-231/IR cells to irradiation. (**J**,**K**) Baicalein sensitized MDA-MB-231/IR cells to Adriamycin (0, 12.5, 25, 50, and 100 nM) and cisplatin (0, 10, 20, 30, and 40 μM) treatment (▬: Adriamycin or cisplatin alone, ▬: Adriamycin or cisplatin with baicalein 10 μM, ▬: Adriamycin or cisplatin with baicalein 20 μM). Asterisks (*) indicate significant differences at *p* < 0.05.

After identifying IFIT2 induction and the decreased stem cell-like properties, we then examined the effect of baicalein on apoptosis in MDA-MB-231/IR cells. Western blot results indicated that the induction of apoptosis by baicalein treatment was accompanied by up-regulation of IFIT2. After treatment with baicalein, the level of IFIT2 increased markedly, by 28.24 ± 0.90 times, while the levels of γ-H2AX increased 5.47 ± 0.91 fold, Bax increased 4.20 ± 0.67 fold, cleaved caspase-3 increased 4.20 ± 0.90 fold, and cleaved PARP increased 5.23 ± 0.95 fold (Figure 6A,B). Cell cycle and annexin V/PI staining also showed an increase in apoptotic cells after baicalein treatment (Figure 6C–E). In particular, JC-1 staining revealed that, as the concentration of baicalein increased, a decrease in red fluorescence, from 98.9 ± 3.51% to 79.8 ± 2.21%, was observed, indicating depolarization of the mitochondrial membrane potential (Figure 6F,G). In addition, combination treatment of baicalein with radiation decreased colony formation and the survival fraction of MDA-MB-231/IR cells compared to baicalein treatment alone (Figure 6H,I). We also tested the effect of combination treatment with several anti-cancer drugs on cell viability. The viability of MDA-MB-231/IR cells treated with both baicalein and Adriamycin (with concentration of 0, 12.5, 25, 50, and 100 nM) or baicalein and cisplatin (with concentration of 0, 10, 20, 30, 40, and 50 μM) was decreased compared to negative control or cells treated with baicalein alone, or anti-cancer drug alone, with combination index (CI) values < 1 (Figure 6J,K, and Supplementary Table S2). These results suggest that baicalein functions as a sensitizer to anti-cancer drugs and radiation in resistant TNBC MDA-MB-231/IR cells.

## 4. Discussion

Treating breast cancer remains challenging despite the various advances in surgery, chemotherapy, and radiotherapy [37]. TNBC is a subtype of breast cancer distinguished by the absence of ER, PR, and HER2, which are potential target molecules in advanced therapies, such as HER2 antigen or hormone treatment [2,3]. Since the TNBC subtype is the most malignant subtype, having many heterogeneous CSCs and limiting the application of advanced therapeutic agents, such as HER2 antigen, androgen receptor modulator, or hormone treatment, the need for the discovery and development of new treatments that can target TNBC is increasing [37,38,39,40].

Phytochemicals are natural compounds derived from plants or fruits. Flavonoids are a subclass of phytochemicals known to have beneficial effects for human health, including anti-cancer effects. The intake of flavonols and flavones, including baicalein, has been associated with a decreased risk of breast cancer [36]. Previous studies on baicalein were mainly focused on inhibiting adhesion, migration, invasion, and cell death in various cancer cells, including breast cancer. The mechanisms of baicalein in cancers involve activation of autophagy through AMP-activated protein kinase (AMPK)-unc-51-like kinase 1 (ULK), cell cycle arrest by CDC2/Cyclin B1, or induction of apoptosis by inhibition of the PI3K/Akt pathway, transforming growth factor (TGF) signaling, myeloid leukemia 1 (Mcl-1), and mammalian target of rapamycin (mTOR) signaling [41,42,43]. Baicalein also overcame TNF-related apoptosis-inducing ligand (TRAIL) resistance by enhancing TRAIL-2 promoter activity through induction of CCAAT/enhancer-binding protein homologous protein (CHOP) in human colon cancer SW480 cells or by increasing expression of TRAIL-2 through ROS induction [25]. However, no study has examined treatment-resistant breast cancer cells using analysis of overall gene expression patterns via transcriptomics, selection of candidate genes, and gene expression analysis before and after treatment with baicalein. In this study, we identified the effect of baicalein on resistant TNBC MDA-MB-231/IR cells generated by repetitive irradiation. Baicalein reversed chemo- and radioresistance, as well as migration, invasion, and stem cell-like properties of the resistant breast cancer cells.

Transcriptomic analysis is a large-scale process using high-throughput techniques to examine RNA molecules of cells [44,45,46]. Comparative transcriptomic studies reveal the different expression patterns between two samples [47]. Analyses of DEGs of MDA-MB-231/IR cells compared to MDA-MB-231 cells revealed genes that were up- or down-regulated by repeated irradiation. DEGs that meet both difference change over four times and fold change over 2.5 times between MDA-MB-231/IR and MDA-MB-231 cells were selected, and 12 genes that are known to play a role in cancer were identified. GO analyses revealed that gene categories that might be associated with chemo- and radioresistance, such as innate immune response, focal adhesion, and the IL-1 receptor binding pathway, were highly activated in MDA-MB-231/IR cells [48,49,50]. Pathway analysis by DAVID revealed that the NF-κB pathway, TNF signaling pathway, and TLR pathway, which interact with signaling pathways that regulate IFIT2, were enriched in MDA-MB-231/IR cells. When we tested the hypothesis that baicalein could reverse the expression of the 12 DEGs, we surprisingly found that only IFIT2 expression was reversed in a dose-dependent manner after treatment with baicalein. Therefore, we propose that the IFIT2 gene is critical for treatment resistance in breast cancer and plays an important role in overcoming resistance with baicalein. Interestingly, when protein interactions were predicted using String 8.0, IFIT1, IFIT2, IFIT3, and TRAF3 were highly interactive, while only IFIT2 was associated with resistance in MDA-MB-231/IR cells (data not shown).

IFIT2, also called interferon-stimulated gene 54 (ISG54), is one of four IFIT proteins, including IFIT1/ISG56, IFIT2/ISG54, IFIT3/ISG60, and IFIT5/ISG58, characterized by a helix-turn-helix motif [51]. It has been reported that IFIT1 and IFIT2 interact with eukaryotic initiation factor 3 (eIF3) to inhibit mRNA translation and recognize viral mRNA structure, which lacks O-methylation [52,53]. In addition to its anti-viral activity, IFIT2 expression has been reported to inhibit cancer cell growth and migration. It has been reported that IFIT2 can interact with cytoskeleton-associated proteins, such as beta-tubulin or cytokeratin 18, and regulate cell mitosis and cell motility while activating protein kinase C (PKC) signaling [54,55]. TNF-α secretion, leading to angiogenesis and metastasis of oral squamous cell carcinoma, can be regulated by IFIT2 depletion [56]. The tumor suppressor promyelocytic leukemia zinc-finger protein (PLZF) was reported to increase expression of IFIT2 in gallbladder cancer [57]. Increased expression of IFIT2 has also been reported during induction of apoptosis by various stimuli, such as interferons, cisplatin, lncRNAs, and miRNAs, in oral cancer cells, osteosarcoma cells, gastric cancer cells, and colorectal cancer cells [56,58,59,60,61]. In human ovarian cancer cells, the down-regulation of IFIT2 was regulated by activation of the Ras/MEK signaling pathway [62]. Induction of IFIT2 has been reported to be involved in apoptosis signaling by disturbing mitochondrial membrane potential in HeLa cells [63]. Here, we confirmed previous reports by conducting JC-1, annexin V/PI, and cell cycle analyses after baicalein treatment. While inducing apoptosis in resistant TNBC MDA-MB-231/IR cells, baicalein increased the mRNA and protein expression IFIT2. In addition, we found that baicalein treatment altered the malignant features of MDA-MB-231/IR cells (e.g., migration, invasion, and stem cell-like properties), and these phenomena were accompanied by reversal of IFIT2 expression. Consistent with these results, TCGA data analysis also revealed that metastasis samples showed lower IFIT2 expression compared to primary tumor and normal tissue, and Kaplan-Meier plots in TNBC patients showed that patients with low IFIT2 expression levels had a higher possibility of relapse (Figure 4D,E).

## 5. Conclusions

In conclusion, we demonstrated for the first time that the phytochemical baicalein reduced stem cell-like properties and induced apoptosis through up-regulation of IFIT2 in chemo- and radioresistant TNBC cells. Baicalein may be a novel phytochemical for inhibiting CSCs and metastasis in TNBC. Our data also suggest that combining conventional therapies with baicalein treatment could enhance therapeutic effects, and we suggest baicalein as a chemo- and radiosensitizer for TNBC patients. Further studies are needed to identify the underlying mechanisms regulated by IFIT2 in resistant breast cancer cells and to investigate the therapeutic effects of IFIT2 on a variety of cancer types possessing high CSCs and expressing resistance.

## Figures and Tables

**Table 1:** Differentially expressed genes (DEGs) in MDA-MB-231/IR cells related to cancer resistance. DEGs are expressed in differences, fold changes (log2), with their functions.

No.	Difference	Fold	Gene	Full Name	Role in Cancer
1	97.57	6.83	AKR1C1	Aldo-keto reductase 1C1	Metastasis
2	79.67	6.64	FTL	Ferritin light chain	Drug resistance
3	77.43	2.92	MK	Midkine	Metastasis
4	62.20	6.01	LG3BP	Galectin-3-binding protein	Anti-differentiation
5	46.92	3.04	GFPT2	Glutamine-fructose-6-phosphate Transaminase 2	Metabolism
6	42.64	3.34	TGFBI	Transforming growth factor beta induced	Metastasis
7	42.35	3.87	AKR1C3	Aldo-keto reductase 1C3	Drug resistance
8	37.59	2.96	CCDC69	Coiled-coil domain-containing 69	Drug resistance
9	14.51	4.13	AKR1C2	Aldo-keto reductase 1C2	Metastasis
10	12.55	+Inf	TWF1	Twinfilin actin binding protein 1	Migration
11	−4.33	−Inf	PKP3	Plakophilin 3	Invasion
12	−85.90	−2.84	IFIT2	Interferon induced protein with tetratricopeptide repeats 2	Apoptosis mediator

Inf, infinity: expression of the indicated gene was not determined.

## Supplementary Materials

The following are available online at https://www.mdpi.com/2072-6643/11/3/624/s1, Figure S1. Cell viabilities and induction of apoptosis in MDA-MB-231 cells and MDA-MB-231/IR cells treated with irradiation or anti-cancer drugs, Table S1. IC_50_ of phytochemical treatments on MDA-MB-231 cells and MDA-MB-231/IR cells. Table S2. CI value of combination treatments on MDA-MB-231/IR cells.

## Abbreviations

ABC	Adenosine triphosphate-binding cassette
ABCG	2ATP-binding cassette super-family G member 2
AKR1C1	Aldo-keto reductase 1C1
AKR1C2	Aldo-keto reductase 1C2
AKR1C3	Aldo-keto reductase 1C3
Akt	Protein kinase B
AMPK	AMP-activated protein kinase
BSA	Bovine serum albumin
CCDC69	Coiled-coil domain-containing 69
CHOP	CCAAT/enhancer-binding protein homologous protein
CI	Combination index
CSCs	Cancer stem cells
DAVID	Database for Annotation, Visualization and Integrated Discovery
DEGs	Differentially expressed genes
DMEM	Dulbecco’s modified Eagle’s medium
DMSO	Dimethyl sulfoxide
DR5	Death receptor 5
eIF3	Eukaryotic initiation factor 3
EMT	Epithelial-mesenchymal transition
ER	Estrogen receptor
FACS	Fluorescence-activated cell sorting
FBS	Fetal bovine serum
FPKM	Fragments per kilobase of transcript per million mapped reads
FTL	Ferritin light chain
GFPT2	Glutamine-fructose-6-phosphate transaminase 2
GO	Gene ontology
HER2	Human epidermal growth factor receptor 2
HR	Hazard ratio
IC50	Inhibitory concentration of 50
IFIT2	Interferon-induced protein with tetratricopeptide repeats 2
IL-1	Interleukin-1
IR	Irradiation
ISG54	Interferon-stimulated gene 54
KAAS	Automatic Annotation Server
KEGG	Kyoto Encyclopedia of Genes and Genomes
LG3BP	Galectin-3-binding protein
Mcl-1	Myeloid leukemia 1
MDR1	Multidrug-resistance protein 1
MK	Midkine
MP	Main population
MRP1	Multidrug-resistance-associated protein 1
mTOR	Mammalian target of rapamycin
MTT	3-(4,5-dimethylthiazol-2-yl)-2,5-diphenyl-tetrazolium bromide
PBS	Phosphate buffered saline
PI	Propidium iodide
PI3K	Phosphatidylinositol 3-kinase
PKP3	Plakophilin 3
PLZF	Promyelocytic leukemia zinc-finger protein
PR	Progesterone receptor
RFS	Relapse-free survival
RIG-1	Retinoic acid-inducible gene-1
RNA-seq	RNA-sequencing
ROS	Reactive oxygen species
RSEM	RNA sequencing by Expectation Maximization
SATB1	Special AT-rich sequence binding protein 1
SP	Side population
STAR	Spliced Transcripts Alignment to a Reference
TCGA	The Cancer Genome Atlas
TGF	Transforming growth factor
TGFBI	Transforming growth factor beta induced
TICs	Tumor initiating cells
TLR	Toll-like receptor
TNBCs	Triple-negative breast cancers
TNF	Tumor necrosis factor
TRAIL	TNF-related apoptosis-inducing ligand
TWF1	Twinfilin actin binding protein 1
ULK	Unc-51-like kinase 1
