# Supplementary material for: Baicalein Suppresses Stem Cell-Like Characteristics in Radio- and Chemoresistant MDA-MB-231 Human Breast Cancer Cells through Up-Regulation of IFIT2
Source: Nutrients. 2019 Mar 14;11(3):624. doi: 10.3390/nu11030624 (PMC6471144; doi:10.3390/nu11030624)
Supplement: Supplementary file 1 [file nutrients-11-00624-s001.pdf]

Supplementary Table S1. IC_{50} of phytochemical treatments on MDA-MB-231 cells and MDA-MB-231/IR cells. Asterisks (*) indicate significant differences at $p < 0.05$.

No.	Group	Phytochemical	IC_{50} (μM)	
			MDA-MB-231	MDA-MB-231/IR
1	Flavone	Apigenin	75.12 ± 1.85	83.11 ± 4.01 *
2		Baicalein	44.25 ± 2.47	38.58 ± 2.86 *
3		Tangeretin	> 200	> 200
4	Flavonol	Kaempferol	> 200	198.52 ± 5.12
5		Myricetin	30.29 ± 1.87	78.61 ± 1.97 *
6		Quercetin	> 200	167.43 ± 2.37 *
7		Rutin	> 200	> 200
8	Phenol	Catechol	185.21 ± 3.69	92.16 ± 1.98 *
9		Chlorogenic acid	> 200	183.66 ± 3.10 *
10		Protocatechuic acid	> 200	> 200

Supplementary Table S2. CI value of combination treatments on MDA-MB-231/IR cells.

Adriamycin (nM)	Baicalein (μM)	CI value	Cisplatin (μM)	Baicalein (μM)	CI value
12.50	10.00	1.88	10.00	10.00	1.02
12.50	20.00	0.82	10.00	15.00	0.79
25.00	10.00	1.70	20.00	10.00	1.08
25.00	20.00	0.57	20.00	15.00	0.83
50.00	10.00	0.60	30.00	10.00	0.94
50.00	20.00	0.41	30.00	15.00	0.61
100.00	10.00	0.42	40.00	10.00	0.54
100.00	20.00	0.40	40.00	15.00	0.32

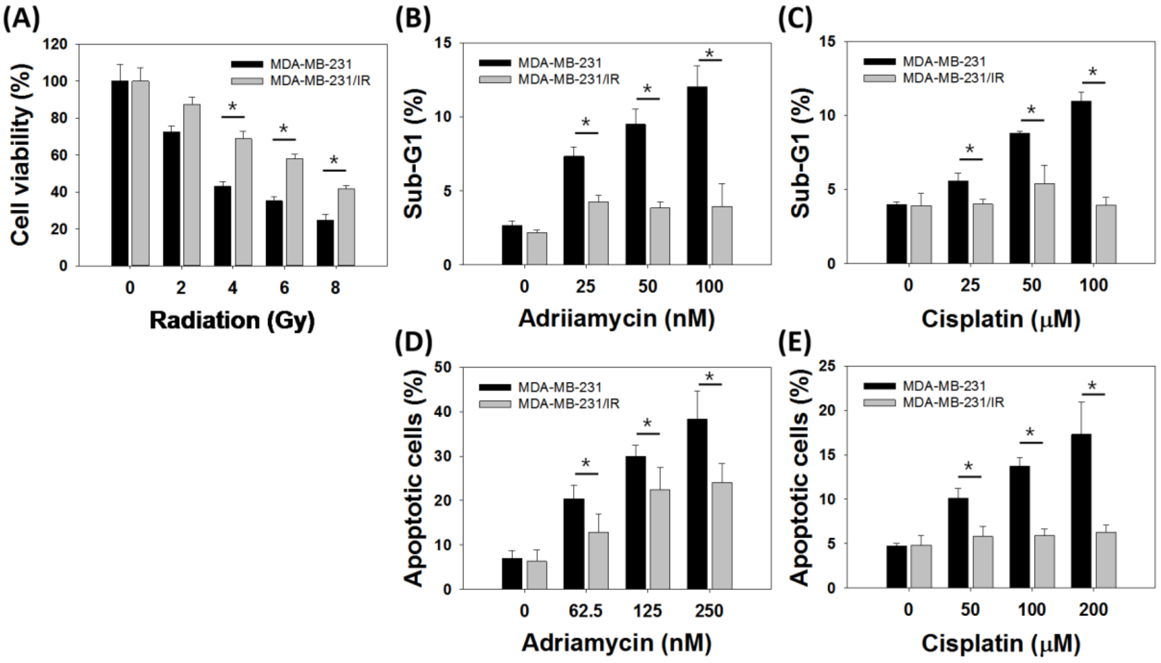

Supplementary Figure S1. Cell viabilities and induction of apoptosis in MDA-MB-231 cells and MDA-MB-231/IR cells treated with irradiation or anti-cancer drugs. (**A**) MTT assay of cells after irradiation. (**B**,**C**) Cells in sub-G1 were analyzed by FACS after Adriamycin and cisplatin treatment for 24 h. (**D**,**E**) Annexin V/PI analysis of cells after Adriamycin and cisplatin treatment for 24 h. Asterisks (*) indicate significant differences at $p < 0.05$.
